# The Effectiveness of Matrix Interventions in Improving Methadone Treatment

**DOI:** 10.5812/ijhrba.8906

**Published:** 2013-03-12

**Authors:** Hossein Eghbali, Mahdi Zare, Arva Bakhtiari, Nader Monirpoor, Alireza Ganjali

**Affiliations:** 1 Department of Clinical Psychology, Islamic Azad University, Qom Branch, Pardisan Town, Qom, IR Iran; 2 The Center for Research and Development in Humanities, SAMT Organization, Tehran, IR Iran; 3 Research Center for Children and Adolescents Health, Zahedan University of Medical Sciences, Zahedan, IR Iran

**Keywords:** Matrix, Anxiety, Depression, Aggression

## Abstract

**Background:**

The treatment of opioid dependence disorder is one of the major problems in medical centers around the world. Although MMT has been the major treatment in last few years in Iran, the existence of relapse before and after detoxification is still high. Methadone treatment has had a very low percentage of complete success.

**Objectives:**

To evaluate the effectiveness of matrix group interventions in improving methadone treatment in the addicted was the main goal of this research.

**Materials and Methods:**

In a semi - experimental design, 24 male patients on the qualification cutoff score for the questionnaire survey (score less than 19 in depression test, and less than 21 in anxiety test) and the diagnosis of opioid dependence according to (DSM – IV) were substituted in two experimental and control groups randomly. At the beginning of the study, after the treatment period and in the follow-up phase (three months after the end of treatment), participants were evaluated by Beck Anxiety Inventory (BAI), Beck Depression Inventory (BDI) and the Anger Questionnaire (AQ), control group with no psychological treatment only took methadone. Data were analyzed using covariance analysis, chi square and Repeated Measures Analysis of Variance.

**Results:**

Results showed that the effect of matrix group interventions on reducing relapse (P < 0.05), increasing the maintenance of treatment (P < 0.01), increasing the treatment compliance, reducing anger, anxiety and depression and methadone dose is more effective than methadone treatment (P < 0.05).

**Conclusions:**

It seems matrix group interventions increase the effectiveness of methadone treatment by reducing the relapse prevention, the dose of methadone and remaining in treatment.

## 1. Background

The treatment of opioid dependence disorder is one of the major problems considered medical centers around the world. Although MMT has been the main treatment in last few years in Iran, the existence of relapse before and after detoxification is still high. Methadone treatment has had a very low percentage of complete success (1). There are some difficulties in MMT. Patients are less successful in the withdrawal of any medications (including methadone). Patients treated by methadone Long-term and in additional time. Some patients fear from withdrawal from MMT (2). The goal of MMT moved from maintenance towards abstinence from all opioid drugs, including methadone (3). But patients remain dependent to opioid or methadone. Although methadone is used in a maintenance treatment and is one of the opioid drugs, there are some kind of lapse and relapse in MMT: 1) Using one type of substances along with methadone, 2) Abusing drugs along with methadone, 3) Relapsing and exiting the MMT, 4) Abusing the high dose of methadone even ingesting two or three times of prescribed dosage, 5) Exiting and entering the treatment periodically with some positive urine test. All types of relapse can be monitored by urinalysis. Urinalysis is often used to monitor relapse and also dose of methadone (4). Results show that patients are less likely to relapse if they participate in an aftercare program and supportive counseling help them for cessation of methadone (5). There is relatively little scientific literature recommending how clinics can best respond to lapses and relapses among patients.

Exiting the MMT before the end of treatment is another problem. About half of those who enter MMT leave the treatment within 12 months and most of them continue to use illicit drugs (6). The duration of treatment, the dose of methadone given and comprehensive non-drug programs are the most important elements of effective treatment (7). It seems that to keep patients in MMT, decreasing the methadone dose, preventing the lapse and relapse in period of methadone therapy, successful detoxification and abstinence need comprehensive non-drug interventions. Some clinics have used reward incentives for continued illicit-drug abstinence, loss of take-home dose and intensive counseling/therapy. This program includes matrix intervention in case of patient problems. Such matrix interventions are used for treatment of amphetamine dependency.

The Matrix Model has been recognized as one of the little evidence based programs for substance abuse disorders and has been listed on the Substance Abuse and Mental Health Services Administration’s (SAMHSA) National Registry of Evidence based Programs and Practices (NREPP). The Matrix approach emphasizes the use of outpatient techniques (8). Treatments focus on:

•Life style changes

•Training in relapse prevention

•Education about dependencies

•Twelve-step facilitation

•Family involvement

Many studies about matrix method have shown the effectiveness of this treatment; for instance alcohol and drug and methamphetamine treatment program (9-12). Matrix treatment is used in Iran by INCAS (Iranian National Center for Addiction Studies) and IRSA (Institute on Psychology and Addiction Sciences) as individual sessions for stimulant dependents Including some self-help training, motivational interview, relapse prevention and cognitive behavioral techniques and will follow in twenty six sessions. Matrix method also has been used in naltrexone Treatment (Rawson, 1998). Using matrix interventions in psycho education and cognitive-behavioral techniques can reduce emotional difficulties, relapse and using the illicit drugs and dose of methadone also increase remaining in treatment and achieving withdrawal of methadone. There are several studies in naltrexone treatment but no research exists about methadone treatment (13-15). Deficits and difficulties of methadone treatment need to be studied, so the aim of this study was to use a new integrated model of matrix interventions consists of common methods of addiction counseling to increase the effectiveness of MMT. The study has been planned and implemented according to the needs of drug addicted to non-drug therapies, particularly cognitive behavioral training, how to deal with craving, lapse and relapse, and family-based intervention strategies and relax therapy techniques to improve energy and positive mood as well.

## 2. Objectives

To evaluate the effectiveness of matrix group, interventions in improving methadone treatment in the addicted was the main goal of this research.

## 3. Materials and Methods

This research was conducted by using a semi-experimental and a control group. the patients of this study were the addicted treated with oral methadone at a center under the supervision of University of Qom for Medical Sciences, Qom, Iran. Participation in the study was restricted to those who, a) Were diagnosed opioid dependency according to DSM–IV–TR, b) Not taking amphetamines merely, c) Were between 20 to 45 years old, d) Had been in the first month of treatment, f) Were male and, g) Not using antipsychotic at the time of study.

Participants in the study were twenty four patients diagnosed with opium dependence, all of which were undergoing methadone maintenance therapy. The study sample was divided randomly into two groups, one receiving methadone treatment with matrix interventions (include: behavioral cognitive therapy in group, family based interventions and relax therapy), and the control group received only methadone treatment. The following instruments were used for data collection.

### 3.1. Beck Depression Inventory (BDI - II)

The short form of Beck Depression Scale comprising of 21 questions, was used to assess depressed mood. This scale covers six of nine DSM-IV criteria for depression and its correlation with the Hamilton Psychiatric Rating Scale for Depression is 0.73, for the MMPI-D depression scale is 0.76 and for Zung self - evaluation depression scale is 0.76. According to Beck (1972), the reliability of this questionnaire using Spearman-Brown is about 0.93. The 21 questions Form of scale examined by PoorShahbaz in Iran (1993) in a sample of 116 person and the correlation coefficient between the scores of two parts with total score achieved between 0.23 - 0.68 and the internal consistency coefficient was 0.85. The reliability of using the Spearman - Brown formula was 0.81 (16).

### 3.2. Beck Anxiety Inventory

This Inventory, which is a useful and valid scale, was used to measure anxiety. The reliability and validity in Iran has been confirmed. Kaviani and colleagues (2009) examined it on clinical population; the validity was 0.7, retest reliability was r = 0.81 and Cronbach's alpha was 0.92 (17).

### 3.3. Buss and Perry Aggression Scale

Buss and Perry questionnaire were used for the assessment of participant's anger. These scale measures five factors; the behavioral aggression, physical and verbal aggression, hostility and non-direct anger. The reliability and validity of this scale in Iran, has been confirmed by Mohammadi (2006) and the Reliability of questionnaire was 0.89 by using Cronbach's alpha, and was 0.78 by using the test and retest and was 0.73 by using half way and also the correlation coefficients was obtained between 0.37 – 0.78 ( P < 0.001) as well, (18).To conduct the study, 24 patients diagnosed with drug dependence were selected based on entry and removal criteria and were assigned in both experimental and control groups randomly. At First, pre-test questionnaires, including depression, anxiety and aggression were filled by both groups. Therapy sessions were based on matrix treatment protocol of IRSA and cognitive behavioral and family training in BDRC and includes 25 sessions of behavioral cognitive therapy in group related to relapse prevention and education of recurrence, seven sessions of family based interventions and eight sessions of relax therapy were conducted. At the end of therapy sessions, both groups filled the questionnaires again. After three months follow up was done. Sessions of behavioral cognitive therapy in group were conducted twice a week and the time of each session was one and a half hours. Family therapy sessions from the first week were conducted once a week and the time of each session was 45 minutes. Relax sessions from the fifth week of treatment were conducted once a week and the time of each session was one and a half hours. The patients in control group did not receive any treatment but drug, and just the patients of test group after being visited by the doctor, received methadone. All patients in both experimental and control groups took urinalysis regularly each week. The daily methadone dosage and all its changes were recorded by treatment staff. At the end of treatments and tests, the results were examined, using methods of descriptive statistics, multivariate analysis of covariance, chi-square and analysis of variance with repeated measures.

## 4. Results

Assessment of demographic and clinical variables showed no significant difference between participants in the two groups (Table 1).

**Table 1.** Descriptive Characteristics of Participants in the Study

Variable	Group 1 ^*^	Group 2 ^**^	χ^2^	df
	Frequency (%)	Frequency (%)		
**Education**				
Elementary	(16.2) 2	(8.3) 1	0.79	2
Secondary and High school	(58.3) 7	(58.3) 7		
Diplomaandhigher education	(25) 3	(33.3) 4		
**Job**				
Employed	(58.3) 7	(66.7) 8	0.5	1
Jobless	(16.7) 5	(33.3) 4		
**Marital status**				
Married	(58.3) 7	(58.3) 7	1.0	1.0
Single	(41.7) 5	(41.7) 5		
**Age**				
22-30	(58.3) 7	(58.3) 7	1.0	2.0
30-40	(25) 3	(25) 3		
40-45	(16.7) 2	(16.7) 2		
**ResidenceStatus**				
Rental	(16.7) 2	(25) 3	0.85	2.0
Personal	(16.7) 5	(41.7) 5		
Father house	(33.3) 5	(33.3) 4		
**Substance**				
Opium	(16.7) 5	(41.7) 5		
Heroine	(58.3) 3	(58.3) 3		
Crack	(25) 3	(33.3) 4		
With amphetamine	(83.3) 2	(97.1) 2		
**Period of use**				
Less than 10 years	(16.7) 7	(33.3) 6	0.833	1.0
**Way of use**				
Smoking	(16.7) 4	(25) 5	0.90	2.0
Oral use	(16.7) 6	(41.7) 5		
Injection	(33.3) 2	(33.3) 2		
Consumption family history	5	4	0.67	1.0
Negative	7	8	-	-

*Group 1: Matrix intervention along with methadone treatment, **Group 2: Methadone treatment

The average duration of dependence was, 9.3 years (SD = 2.87) on matrix interventions group, and 10.16 years (SD = 3.34) in the methadone group. The kind of substance and the way of its using was not significantly different in both groups (Table 2).

**Table 2.** The Contents of Interventions and Sessions

Session	Type of Intervention, Content and Techniques
	**Behavioral-cognitive Therapy in Group**
**1**	
Contracts	Introduction, Group principles, Motives, Objects and Commitment
**2-3**	
Drug training	Process of MMT, Methadone, Why and How, Cost-benefit of avoiding and Dependency, Complete avoiding
**4-6**	
Craving	Triggers, External and Internal triggers, Related behaviors and Believes,Craving Management
**7-9**	
Common Difficulties	Stages of treatment, Emotions and Symptoms, Mistrust, Energy reducing, Logical errors
**10-12**	
Relapse prevention (1st)	A-B-C, Being bored, Feelings of guilt, Relapse activators and prevention activities,Addictive behaviors,Drug abusing
**13-17**	
Believes	The vertical arrows, SUD, Cognitive maps, List of beliefs and evaluation, Review and opposition to defaults
**18-20**	
Relapse prevention (2nd)	Emotions changing, Change the schemas, Preventive activities
**22-24**	
Change of functions	Identification of negative reactions and behavioral patterns, Activating events, Behavior Dysfunctional behaviors and alternative behaviors, Steps for behavioral change, Motivational factors, Self-regulation
**25**	
Problem solving	Problem solving steps, Rational and irrational beliefs and strategies in problem solving
	**Family Oriented Interventions**
**1-2**	
Treatment	Process of treatment, Medical model approach, Family duties
**3-4**	
Mistrust	Appropriate way to interaction, How to criticize, Creating and maintain the self-concept, Management of relapse
**5-7**	
Relations	Examine the relationships and roles, Methods of problem solving, Empathic perception, Communication obstacles
	**Relax Therapy**
**1-4**	
Body practices	Physical exercise, Concentration, Diaphragmatic breathing
**5-7**	
Mental practices	Elementary meditation, Awareness of feelings, Reinforce the good feelings

*P < 0.01, **P < 0.05

In the first, second, third and fourth research hypotheses, MANCOVA with pretest control, the effectiveness of matrix interventions was shown by reducing anxiety and depression (*P* < 0.05), anger and methadone daily dosage (*P* < 0.01) (Table 3).

**Table 3.** Results of Multivariate Covariance Analysis

Variable	Pretest-posttest			Pretest-follow up		
	F	df	μ^2^	F	df	μ^2^
**Anxiety**						
Covariate	6**	1	0.25	5.76**	1	35
Treatment effect	15.16**	1	0.457	15.78**	1	0.21
Residual error		18			18	
**Depression**						
Covariate	9.32*	1	0.34	8.78*	1	0.35
Treatment effect	6.78**	1	0.27	6.12*	1	0.29
Residual error		18			18	
**Anger**						
Covariate	3.57*	1	0.656	3.06*	1	0.66
Treatment effect	71*	1	0.8	64*	1	0.786
Residual error		18			18	
**Daily methadone use**						
Covariate	10.5*	1	0.854	9.7*	1	0.35
Treatment effect	35.7*	1	0.665	35.4*	1	0.8
Residual error		18			18	

^*^*P < 0.01, **P < 0.05

Due to the test, matrix interventions reduced depression, anxiety and anger in addicts. Analysis of variance with repeated measurements used for Group 1 (matrix interventions) to control the pre-test anxiety, depression and anger scales, methadone dosage changes. As (Table 4), indicates matrix interventions had a significant effect on all measured variable.

**Table 4.** Results of Analysis of VarianceWith Repeated Measures

	μ^2^	P Value	F
**Anxiety**	37.6	0.001	0.788*
Residual error			**
**Depression**	20.5	0.001	0.651
Residual error			**
**Anger**	37	0.01	0.16*
Residual error		18	**
**Daily methadone use**	35	0.006	0.632
Residual error			**

^*^df = 22**, df = 2*

Assessment of the effectiveness of matrix interventions on treatment compliance; based on remaining in treatment and the period of methadone use, indicated significant χ^2^ (*P* < 0.05 and χ^2^ = 6.92).

The results shows a significant difference between Group one and two in the period of methadone use. Average remaining in treatment in patients of Group one was 195 days (SD = 7.35) and in patients of group two was 132 days (SD = 9.61). Manova indicated Average remaining in treatment in group one (matrix intervention) is more (F = 9.87 and *P* < 0.01).

Also results indicated a significant difference in relapse rates between the two groups at posttest (*P* < 0.05 and χ^2^ = 3.6), also at follow up (*P* < 0.05 and χ^2^ = 4.2). At the posttest relapse, the rate in matrix intervention group was one patient or 3.8% and in methadone treatment group was five patients, or 7.41%. Also at the end of follow up relapse, the rate in matrix intervention group was three patient or 25% and in methadone treatment group was eight patients, or 7.66%.

## 5. Discussion

The purpose of present study was to investigate the effectiveness of matrix interventions included behavioral cognitive therapy in group, family-based interventions and relax therapy in improving methadone treatment. Findings showed that matrix interventions, increases effectiveness of treatment and is more effective in relapse prevention compared to methadone treatment alone. Other results showed that matrix interventions is more effective in reducing anxiety, depression, anger, and daily dose of methadone and increasing the treatment compliance and remaining in treatment compared to methadone treatment alone. The findings of study correspond with those of Obert, London and Rawson (11), with matrix interventions in the addicted to psychotropic substances, alcohol and opiates is more effective than drug or usual treatment. In study of Azizi, Borjali and Golzari (13), the effectiveness of emotion regulation training and cognitive therapy on Emotional Addictional Problems and Treatment compliance was higher than naltrexone group. The similar Results were obtained in present study. In explaining the effectiveness of matrix interventions on mood and anxiety problems, it can be concluded that treatment process training, influence believes, concerns about relapse and treatment process and problem solving skill has been effective to reduce patients' mood problems. Some difficult parts of addiction treatment can be overcome by reviewing the main conflicts in the family and consequently it will reach the treatment agreement in family therapy, and enhance coordination and cooperation in family to relapse prevention. While relax therapy techniques such as diaphragmatic and energizing breathing had obvious effect on the energy and the motivation of patients. Various researches refer to anger impulses in the addicted and importance of aggression management in relapse prevention such as Schreiber *et al*. (19), Huber (10), Rawson (8). In one study group, cognitive therapy decrease relapse prevention and treatment-retain rate and abstinence rate turn high (20). In study, urine testing and contingency programs increase the abstinence (21). In present study, Decrease of relapse and increase of treatment remaining were the same and made the MMT more effective.

It seems that in present study behavioral cognitive therapy, emotional awareness and improved family relationships reduce anger scale scores in patients and helped the relapse prevention. The findings of study in reducing recurrence and increasing treatment compliance correspond with those of Larimer *et al*. (22); Witkiewitz *et al*. (23); Hunt *et al*. (24), Havard *et al*. (25), and Hufford *et al*. (26). In this study, we tried to solve some problems of methadone treatment by using relax therapy such as agitation, nervousness, lack of concentration, reduction of energy, Unpleasant feelings and pains. These techniques Along with family and behavioral cognitive interventions, motivational techniques, problem solving, social support, adaptation without drug use and use of available resources presented to methadone treatment, remaining in treatment and prevent of relapse. The limitations of this study were short term follow-up, use of one therapist, a low number of participants and lack of research on matrix interventions in Iran for comparison. More accountability flexibility can be obtained by larger samples, comparing different treatments and control of other treatment indicators.
